# S100B chaperone multimers suppress the formation of oligomers during Aβ42 *aggregation*

**DOI:** 10.3389/fnins.2023.1162741

**Published:** 2023-03-21

**Authors:** António J. Figueira, Joana Saavedra, Isabel Cardoso, Cláudio M. Gomes

**Affiliations:** ^1^BioISI–Instituto de Biosistemas e Ciências Integrativas, Faculdade de Ciências, Universidade de Lisboa, Lisbon, Portugal; ^2^Departamento de Química e Bioquímica, Faculdade de Ciências, Universidade de Lisboa, Lisbon, Portugal; ^3^i3S–Instituto de Investigação e Inovação em Saúde, Universidade do Porto, Porto, Portugal; ^4^IBMC–Instituto de Biologia Molecular e Celular, Universidade do Porto, Porto, Portugal; ^5^ICBAS–Instituto de Ciências Biomédicas Abel Salazar, Universidade do Porto, Porto, Portugal

**Keywords:** molecular chaperones, protein aggregation, amyloid-β oligomers, aggregation kinetics and mechanism, amyloid beta (1–42)

## Abstract

Extracellular aggregation of the amyloid-β 1–42 (Aβ42) peptide is a major hallmark of Alzheimer’s disease (AD), with recent data suggesting that Aβ intermediate oligomers (AβO) are more cytotoxic than mature amyloid fibrils. Understanding how chaperones harness such amyloid oligomers is critical toward establishing the mechanisms underlying regulation of proteostasis in the diseased brain. This includes S100B, an extracellular signaling Ca^2+^-binding protein which is increased in AD as a response to neuronal damage and whose holdase-type chaperone activity was recently unveiled. Driven by this evidence, we here investigate how different S100B chaperone multimers influence the formation of oligomers during Aβ42 fibrillation. Resorting to kinetic analysis coupled with simulation of AβO influx distributions, we establish that supra-stoichiometric ratios of dimeric S100B-Ca^2+^ drastically decrease Aβ42 oligomerization rate by 95% and AβO levels by 70% due to preferential inhibition of surface-catalyzed secondary nucleation, with a concomitant redirection of aggregation toward elongation. We also determined that sub-molar ratios of tetrameric apo-S100B decrease Aβ42 oligomerization influx down to 10%, while precluding both secondary nucleation and, more discreetly, fibril elongation. Coincidently, the mechanistic predictions comply with the independent screening of AβO using a combination of the thioflavin-T and X-34 fluorophores. Altogether, our findings illustrate that different S100B multimers act as complementary suppressors of Aβ42 oligomerization and aggregation, further underpinning their potential neuroprotective role in AD.

## Introduction

Aggregation of the disordered amyloid-β peptide (Aβ) into extracellular plaques constitutes a major hallmark in Alzheimer’s disease (AD) neurodegeneration (Hardy and Higgins, 1992; Knowles et al., 2014; Selkoe and Hardy, 2016). Indeed, the conversion of monomeric Aβ into mature amyloid fibrils involves a complex self-assembly process that leads to the formation of transient and off-pathway soluble/fibrillar species (Kayed et al., 2007), globally designated as Aβ oligomers (AβO) (Michaels et al., 2018). Increasing evidence suggests that AβO, rather than mature amyloid fibrils, are the key AD etiological drivers responsible for disease progression (Holtzman et al., 2011; Benilova et al., 2012). Various populations of AβO were reported to trigger multiple deleterious events that ultimately contribute to neuronal loss and cognitive decline (Cline et al., 2018). Examples include tau hyperphosphorylation and missorting (Ma et al., 2009; Schützmann et al., 2021), oxidative stress (De Felice et al., 2007; Wang et al., 2014), metallostasis dysregulation (Lazzari et al., 2015; Cristóvão et al., 2016), cell membrane damage (Williams et al., 2011), axonal transport impairment (Decker et al., 2010), synaptic receptor redistribution (Lacor et al., 2007), and astroglia activation (Heneka et al., 2015; Forloni and Balducci, 2018). In particular, reactive astrocytes surround Aβ proteinaceous aggregates (Kato et al., 1998) and prompt local neuroinflammation by secreting several alarmins into the synaptic milieu (Li et al., 2011).

This is the case of the small homodimeric (2 × 10.7 kDa) Ca^2+^-binding S100B protein (Mrak and Griffinbc, 2001; Donato et al., 2009), which is upregulated in AD (Marshak et al., 1992) and is known to contribute to the late neuroinflammatory response (Cuello, 2017; Hagmeyer et al., 2019). Apart from its pro-inflammatory role, we have recently unveiled that the Ca^2+^-bound form of dimeric S100B inhibits the *in vitro* aggregation and toxicity of the Aβ forty two amino-acid variant (Aβ42) (Cristóvão et al., 2018, 2020) and the microtubule-associated protein tau (Moreira et al., 2021), thus acting as a neuroprotective holdase-type chaperone. Not only, and although mainly present in the brain as a homodimer, S100B is also found as higher order functional oligomers such as octamers, hexamers and tetramers (Ostendorp et al., 2007), the latter described as suppressors of Aβ42 aggregation (Figueira et al., 2022). Indeed, we recently established that S100B tetramerization spawns an extended hydrophobic surface which is formed by the lateral juxtaposition of homodimer C-terminal helices and whose solvent accessibility is independent of Ca^2+^ binding. This novel regulatory cleft leads to a significant increase of Aβ42 anti-aggregation activity, also observable in the apo-form of tetrameric S100B (Figueira et al., 2022). Structural nuclear magnetic resonance (NMR), immunogold labeling electron microscopy and computational studies show that dimeric and tetrameric S100B target monomeric and fibrillar Aβ42 conformers (Cristóvão et al., 2018; Rodrigues et al., 2021), which indicates a modulation of Aβ42 primary nucleation, fibril elongation and, particularly, fibril catalyzed secondary nucleation of monomers into small aggregates (Arosio et al., 2016; Cristóvão et al., 2018). Given that secondary nucleation constitutes a major source of on-pathway Aβ42 oligomers (Arosio et al., 2015; Tornquist et al., 2018), aggregation suppressors able to impair this microscopic reaction step result in a significant depletion of AβO formed along the aggregation process, thus being attractive candidates for disease-modifying approaches. This is the case of the Brichos chaperone domain (Cohen et al., 2015; Chen et al., 2020), numerous bexarotene derivatives (Chia et al., 2018), and the anti-Aβ Aducanumab antibody (Linse et al., 2020). Driven by this evidence, we here investigate if dimeric and tetrameric S100B, the two most abundant non-covalent S100B multimers present in the human brain (Ostendorp et al., 2007), could exert an analogous effect over AβO generation.

While several biophysical approaches are available for the kinetic monitoring of mature amyloid fibrils (Biancalana and Koide, 2010), the detection of intermediate species is particularly challenging due to the conformational heterogeneity and residual amounts of AβO. Although experimental methods based on ELISA immunodetection (Aprile Francesco et al., 2020), mass spectrometry and tritium scintillation (Michaels et al., 2020) were previously employed to timely screen pre-fibrillar AβO populations, a simpler approach builds in the kinetic simulation of the nucleation rates throughout Aβ aggregation (Chia et al., 2018). This represents a robust and relatively simple method to estimate fibrillar AβO distributions exclusively based in the rate constants drawn from thioflavin-T (ThT) aggregation kinetics assays (Gade Malmos et al., 2017). Interestingly, some amyloid binding fluorophores are reported to detect ThT negative conformers early in the aggregation reaction. Indeed, fluorescent dyes such as the p-FTAA luminescent conjugated oligothiophene (Klingstedt et al., 2011), the Congo red derivative X-34 (Barton et al., 2019) and the molecular rotors DCVJ (Nagarajan and Lapidus, 2017) and ThX (Needham et al., 2020), were described to have enhanced selectivity for oligomers in particular aggregation systems. In this study, we take advantage of these properties and combine different amyloid fluorescent dyes to monitor Aβ42 aggregation kinetics and infer on the extent of formed oligomers through differential analysis. Interestingly, we find this to correlate adequately with AβO distributions determined independently through mechanistic analysis.

## Results and discussion

### S100B multimers modulate multiple pathways governing Aβ42 aggregation

**FIGURE 1:**
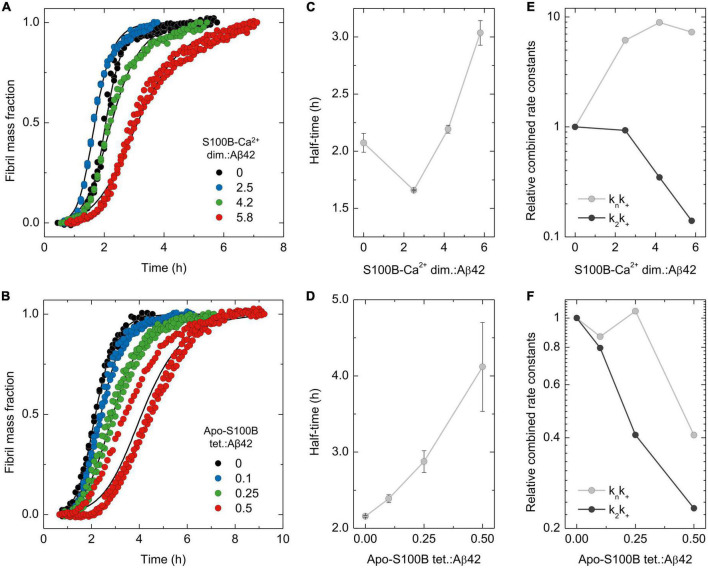
Analysis of dimeric and tetrameric S100B effect over Aβ42 aggregation mechanisms. Normalized traces of ThT-monitored aggregation of monomeric Aβ42 [6 μM in panel **(A)** or 2 μM in panel **(B)**] in the presence of increasing molar ratios of **(A)** S100B-Ca^2+^ dimer:Aβ42 (0–5.8) or **(B)** apo-S100B tetramer:Aβ42 (0–0.5) with the **(C,D)** respective values of aggregation half-times (t_1/2_). Solid lines depict global fits of each curve obtained by varying the rate constants of primary (*k*_*n*_*k*_+_) and secondary (*k*_2_*k*_+_) Aβ42 aggregation pathways, with the relative values of each combined rate constant plotted as a function of S100B **(E)** dimer and **(F)** tetramer ratios. In all cases, error bars represent standard deviation of three experiments.

To establish the impact of S100B on the generation of Aβ42 oligomers, we started by characterizing the mechanistic properties contributing for S100B anti-aggregation activity in the Ca^2+^-bound dimeric and apo-tetrameric states. For this, we employed Aβ42 thioflavin-T (ThT) monitored aggregation assays coupled with global fitting analysis, which provides information about the microscopic mechanisms accounting for the macroscopic kinetic traces (Figures 1A–F).

Complying with our prior observations, we observe that the calcium bound form of dimeric S100B (S100B-Ca^2+^) suppresses Aβ42 aggregation at supra-stoichiometric conditions (Figures 1A, C). Global fitting of the kinetic traces implies that Aβ42 aggregation secondary pathways, a combination of the secondary nucleation and elongation rates constants (*k*_2_*k*_+_), are properly suppressed by an excess of S100B-Ca^2+^ dimer (Figure 1E). This inhibitory behavior is indeed consistent with the multiple Aβ42 conformers previously shown to interact with S100B-Ca^2+^, and which include monomers, oligomers and fibril surfaces (Cristóvão et al., 2018). Primary pathway rates (*k*_*n*_*k*_+_) are contrastingly enhanced in the presence of the chaperone (Figure 1E), settling with the mild acceleration of Aβ42 fibrillation observed at lower S100B:Aβ42 ratios (Figure 1C). In fact, an increase over primary nucleation rates was reported to occur in other amyloid suppressors (Linse et al., 2020; Poska et al., 2020), particularly for proteins exposing aggregation-prone surfaces as Ca^2+^-bound S100B (Cristóvão et al., 2021). Such effect yields, however, a negligible perturbation over the amounts and formation rates of intermediate Aβ42 oligomers, as previously characterized (Arosio et al., 2015). To gain further insights in the specific mechanisms influenced by S100B-Ca^2+^, we performed global fitting of the kinetic data but, this time, allowing only a specific rate constant (*k*_*n*_, *k*_*2*_ or *k*_*+*_) to be the free fitting-parameter transversely to all conditions (Supplementary Figures 1A–C). This semiempirical analysis reveal that the experimental data is best described by a specific reduction of the surface catalyzed nucleation rate constant *k*_*2*_ (Supplementary Figure 1B). Consequently, results support that dimeric S100B-Ca^2+^ effect over Aβ42 secondary paths is largely caused by an impairment of surface catalyzed nucleation events.

Using an analogous procedure, we set out to investigate the mechanistic aspects driving the activity of tetrameric apo-S100B under sub-stoichiometric conditions. We tested tetrameric S100B in a Ca^2+^ free state, as this allow us to selectively investigate the catalytic activity of the novel extended cleft present in tetrameric S100B (Figueira et al., 2022). As reported, tetrameric apo-S100B displays an enhanced anti-aggregation activity with respect to dimer, being able to delay Aβ42 fibrillation even at sub-molar ratios (Figures 1B, D). Global fitting analysis reveal a simultaneous suppression of both Aβ42 combined aggregation rate constants, with a higher impact on *k*_2_*k*_+_values (Figure 1F). As this scenario is compatible with an inhibition of any Aβ42 aggregation micro-steps, we again performed global fitting allowing variations of each individual rate constant (Supplementary Figures 1D–F). We observed that only selective perturbations in secondary nucleation (*k*_*2*_) and elongation rate (*k*_*+*_) constants lead to appropriate explanation of the experimental kinetic traces (Supplementary Figures 1E, F). Therefore, we are guided to conjecture that the decrease over primary and secondary pathway rate constants results from a concomitant and more balanced inhibition of secondary nucleation and fibril elongation.

### Dimeric and tetrameric S100B preferentially suppress Aβ42 secondary nucleation and minimize oligomer generation

**FIGURE 2:**
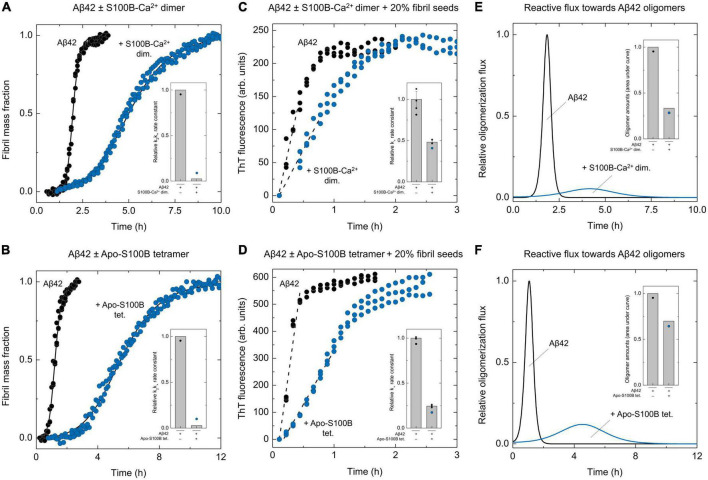
Effect of S100B dimer and tetramer over Aβ42 oligomerization flux distributions. Normalized kinetic traces and global fits (solid lines) of unseeded ThT-monitored aggregation of Aβ42 [6 μM in panel **(A)** or 4 μM in panel **(B)**] in the absence and presence of **(A)** dimeric S100B-Ca^2+^ (S100B:Aβ42 = 5.8) or **(B)** tetrameric apo-S100B (S100B:Aβ42 = 0.5) with indication of the relative secondary pathways combined rate constants (*k*_2_*k*_+_, insets). Kinetic traces of high-seeded (20% Aβ42 fibrils). ThT-monitored aggregation of Aβ42 [6 μM in panel **(C)** or 4 μM in panel **(D)**] in the absence and presence of **(C)** dimeric S100B-Ca^2+^ (S100B:Aβ42 = 5.8) or **(D)** tetrameric apo-S100B (S100B:Aβ42 = 0.5), depicting the plot regions used for linear fits (dashed lines) and the calculated relative elongation rate constants (*k*_*+*_, insets). Error bars represent standard deviation of three experiments. Relative Aβ42 oligomerization flux/nucleation rate distributions (solid lines) in the absence/presence of **(E)** dimeric S100B-Ca^2+^ (S100B:Aβ42 = 5.8) or **(F)** tetrameric apo-S100B (S100B:Aβ42 = 0.5) and total amounts of oligomers calculated by integration of each distribution in respect to time (insets).

To quantitatively dissect the partial contributions of secondary nucleation and fibril elongation for the observed *k*_2_*k*_+_ depletions, we then performed experiments at high seeding conditions (20% of Aβ42 fibrils), and employing the highest S100B:Aβ42 ratios assayed (Figures 2A–F). Indeed, under these conditions Aβ42 aggregation is completely dominated by elongations events, and the initial slope of the resulting kinetic profile can be used to estimate the correspondent elongation rate constants *k*_*+*_ (Meisl et al., 2014). Results show that unlike the unseeded condition, in which dimeric S100B-Ca^2+^ strongly reduces to <5% the combined rate constant *k*_2_*k*_+_ (Figure 2A), under a high seeding kinetic regime the *k*_*+*_ reduction is limited to 50% (Figure 2C). We thus conclude that the S100B-Ca^2+^ dimer effect on the apparent *k*_2_*k*_+_ value is mainly caused by an inhibition of Aβ42 nucleation on fibril surfaces. With respect to tetrameric apo-S100B, high-seeding assays denote a more vigorous inhibition over the mechanism of fibril elongation, associated with a 75% *k*_*+*_ reduction (Figures 2B, D). Nonetheless, and despite this stronger effect, we find that the *k*_2_*k*_+_ depletion is still mostly prompted by an impairment of Aβ42 secondary nucleation.

Given the pivotal role of secondary nucleation in AβO catalytic cycle (Tornquist et al., 2018), we posited that the activity of S100B multimers could result in a depletion of nucleation units/oligomers formed throughout Aβ42 fibrillation. To test this hypothesis, the determined relative *k*_*+*_ values were used to derive primary nucleation (*k*_*n*_) and secondary nucleation (*k*_*2*_) individual rate constants, which in turn were employed to simulate Aβ42 oligomerization flux distributions (Figures 2E, F). Simulations reveal that an excess of S100B-Ca^2+^ dimer results in a substantial suppression of Aβ42 oligomers formation during the fibrillation process. We observe a drastic 95% reduction in the maximum reactive flux toward Aβ42 oligomers and peak integration reveals that the number of AβO is decreased by 70% in the presence of S100B (Figure 2E). A similar effect is noticed for the apo-S100B tetramer under sub-stoichiometric conditions, with the maximum rate of Aβ42 oligomer formation being diminished down to 10% (Figure 2F). Nonetheless, and contrastingly to the activity of dimeric S100B, we find that the tetramer has a more modest 30% reduction effect in AβO total amounts. This is, however, in line with the improved inhibitory effect of the S100B tetramer over fibril elongation, as this mechanism constitutes the major reactive path by which nucleated aggregates can be converted into more matured fibrils (Staats et al., 2020).

Taken together, kinetic assays show that both dimeric and tetrameric S100B strongly minimize the formation of oligomers during Aβ42 fibrillation. Such inhibitory profile arises from a preferential targeting of secondary nucleation, which causes a subsequent redirection of Aβ42 aggregation toward elongation events.

### Estimation of Aβ42 oligomer distributions from differential fluorescence analysis

**FIGURE 3:**
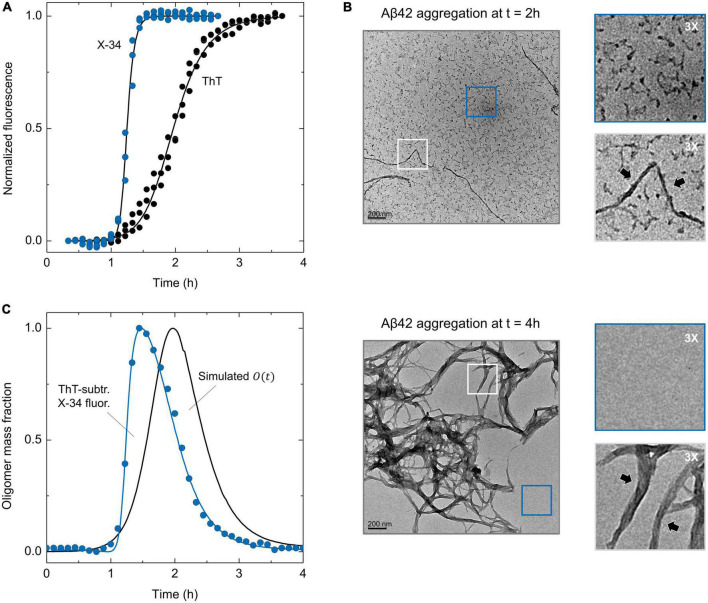
ThT and X-34 differential fluorescence analysis of Aβ42 aggregation and TEM imaging of X-34 positive oligomers. **(A)** Comparison of Aβ42 (6 μM) aggregation kinetics monitored by the ThT and X-34 fluorophores. **(B)** Representative transmission electron microscopy images of Aβ42 aggregation at the time-points of (top) 2 and (bottom) 4 h. Gray and blue insets represent three-times ampliation of Aβ42 amyloid-like fibrils and small oligomers/background, respectively. Black arrows pinpoint Aβ42 amyloid fibrils. **(C)** Temporal evolution of ThT subtracted X-34 positive species (average values, *n* = 3) overlayed with Aβ42 oligomer mass fraction progression [*O*(*t*)] calculated from aggregation rate constants (black solid line), portraying a significant intersection between experimental and simulated kinetics. See Supplementary Figures 3A–C for details on the mathematical formalism employed.

To gain further experimental insight about the effect of S100B over AβO populations, we resorted to a combination of the amyloid-binding fluorophores ThT and the Congo red derivative X-34. X-34 was reported to detect early amyloidogenic species in addition to mature amyloid fibrils (Rodriguez Camargo et al., 2021), including globular oligomers and small curly filaments (Barton et al., 2019). In agreement, when X-34 is employed to monitor the aggregation of Aβ42, a kinetic profile with a shorter lag phase and earlier plateau is observed, in respect to that obtained on a ThT-monitored aggregation (Figure 3A). To rule out that the possibility that either X-34 or DMSO (the dye solvent) might be causing acceleration of Aβ42 aggregation, we performed appropriate controls using increasing dye (1, 2 and 5 μM) and solvent concentration (1% in volume), which yielded superimposable kinetic traces (Supplementary Figures 2A, B). We also performed scaling exponent analysis of Aβ42 aggregation monitored with X-34 and obtained a scaling exponent (γ) of–1.26 ± 0.20, which indicates that, similarly to what is obtained with ThT (Cohen et al., 2013; Meisl et al., 2016), X-34 monitored Aβ42 aggregation kinetics follows a mechanism dominated by secondary nucleation (Supplementary Figures 2C, D). Therefore, the early increase in X-34 fluorescence observed during Aβ42 aggregation suggests that this fluorophore is effectively detecting Aβ42 oligomers. To obtain additional evidence we employed transmission electron microscopy (TEM) for morphological analysis of Aβ42 species at different time points of aggregation. At *t* = 2 h, which corresponds to the ThT half-time and to the X-34 plateau phase, we observed mostly small (<50 nm) Aβ42 aggregates in addition to sparse fibrillar materials (Figure 3B top). On the other hand, at *t* = 4 h, which corresponds to the ThT-plateau phase, we observed essentially mature Aβ42 fibrils with a high-level of self-association but no small oligomers (Figure 3B bottom). Therefore, TEM imaging confirms that X-34 detects early Aβ42 aggregates that are on pathway to the formation of mature amyloid fibrils. This result prompt us to establish a procedure through which the combined monitoring of Aβ42 aggregation kinetics using both X-34 and ThT, might result in a straightforward experimental estimate for formed AβO. This approach is based on the premise that the algebraic subtraction between X-34 and ThT normalized kinetic traces will yield a fair estimate of AβO, allowing a straightforward experimental estimate for the evolution of AβO during Aβ42 aggregation. To evaluate such possibility, we compared the mechanistically derived mass progression of Aβ42 oligomers [*O*(*t*)] obtained from the ThT-aggregation rate constants (Supplementary Figure 3) with the computed kinetic profile obtained from the algebraic subtraction between X-34 and ThT normalized kinetic traces (Figure 3C). Strikingly, the comparison of this experimentally derived trace with the simulated progression of Aβ42 oligomers revealed a significant overlap between the two distributions, whose maxima differ by only by ∼ 0.5 h. Given the complex and dynamic nature of AβO (Michaels et al., 2020), this fluorescence kinetics difference method provides a fairly reasonable estimate of these species obtained from straightforward kinetic experiments.

### Effect of dimeric and tetrameric S100B on X-34 positive Aβ42 oligomers

**FIGURE 4:**
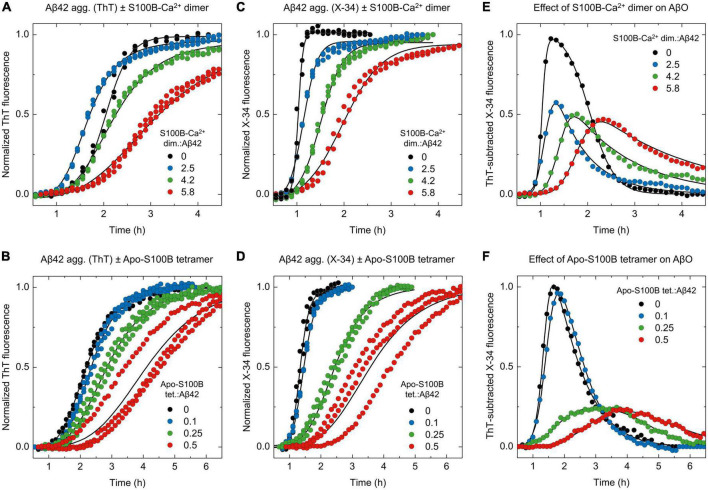
ThT and X-34 differential fluorescence analysis support the S100B dimer and tetramer suppressor effect over Aβ42 oligomeric species. Normalized kinetic traces of **(A,B)** ThT or **(C,D)** X-34 monitored aggregation of monomeric Aβ42 [6 μM in panels **(A,C)** or 2 μM in panels **(B,D)**] in the presence of increasing molar ratios **(A,C)** of S100B-Ca^2+^ dimer:Aβ42 (0–5.8) or **(B,D)** apo-S100B tetramer:Aβ42 (0–0.5). ThT subtracted X-34 kinetic traces (average values, *n* = 3) of all **(E)** S100B-Ca^2+^ dimer and **(F)** apo-S100B tetramer concentrations assayed. Non-normalized kinetic traces are depicted in Supplementary Figure 4.

Once we established the screening of AβO using X-34, the effects of S100B-Ca^2+^ dimer and apo-S100B tetramer were examined (Figures 4A–F; Supplementary Figure 4). Firstly, we monitored Aβ42 aggregation employing the two fluorophores side-by-side at increasing supra-stoichiometric ratios of S100B-Ca^2+^ dimer (Figures 4A, C). The evolution of X-34 positive non-thioflavin Aβ42 oligomers was again computed by subtracting each X-34 kinetic to the corresponding ThT trace, for a given S100B concentration. Corroborating the mechanistic predictions, experimental outcomes reveal that in addition to delay the time required for AβO emergence, an excess of dimeric S100B-Ca^2+^ suppresses the maximum formed amounts of X-34 positive Aβ42 oligomers down to 45%, in a concentration dependent manner (Figure 4E). This quantity was reduced to 65% even at the lowest ratio assayed, suggesting that the S100B-Ca^2+^ dimer has an anti-oligomerization effect even at such molar proportions. Correspondingly, the establishment of more mature and structured Aβ42 species that are simultaneously positive for X-34 and ThT also provides evidence for an S100B-induced redirection of Aβ42 aggregation toward elongation events. A similar phenomenon was actually verified in other nucleation suppressor chaperones, whose activity elicited the formation of longer and more ordered Aβ amyloid fibrils, as inferred by bio-imaging techniques (Cohen et al., 2015; Limbocker et al., 2019).

Lastly, we exploited the X-34 detection of Aβ42 oligomers to evaluate the effect of apo-S100B tetramer, at similar sub-stoichiometric conditions (Figures 4B, D). ThT-subtracted X-34 kinetic traces indicate that tetrameric S100B drastically inhibit the generation of X-34 positive AβO down to 25%, an effect that is produced even at a apo-S100B tetramer. Aβ42 molar ratio of 0.25 (Figure 4F). Indeed, we notice a close overlap between X-34 and ThT kinetics in the presence of tetrameric apo-S100B at molar proportions ≥ 0.25, again suggesting a predominance of more structured thioflavin-T positive species throughout Aβ42 fibrillation. We thus conclude that as postulated by a mechanistic-based analysis, Aβ42 binding by tetrameric S100B affords a potent oligomer suppressor effect which occurs at sub-stoichiometric concentrations and in a Ca^2+^ independent fashion.

## Conclusion

Whereas a number of molecular and chemical chaperones were described to halt *in vitro* and *in vivo* Aβ42 fibrillation, only a limited set is able to target the specific microscopic mechanisms responsible for the generation of neurotoxic oligomers (Arosio et al., 2016; Mannini and Chiti, 2017). Noteworthy, previous work has shown that anti-oligomerization chaperones provide neuroprotection in Aβ-challenged murine brain models (Cohen et al., 2015; Chen et al., 2020), thus making such proteostasis regulators attractive inspirations for prospective AD therapies. Here we resorted to a combination of mechanistic and kinetic approaches to investigate the hypothesis that the S100B synaptic chaperone could also be able to minimize the formation of Aβ42 oligomers on-pathway to fibril formation.

**FIGURE 5:**
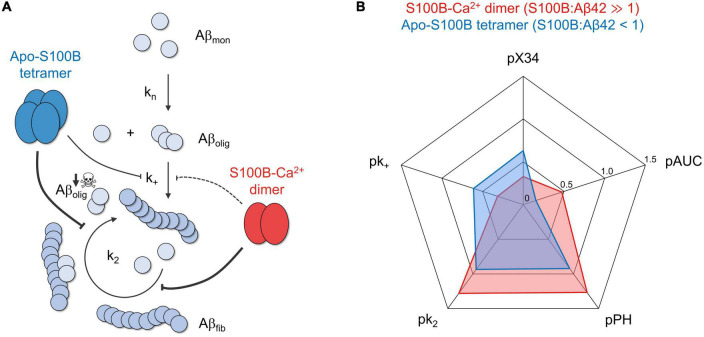
The chaperone activity of dimeric and tetrameric S100B suppresses the generation of intermediate oligomers during Aβ42 aggregation. **(A)** S100B dimer and tetramer suppression of Aβ42 oligomerization based on the discreetly distinct inhibition of multiple aggregation micro-steps and the preferential targeting of fibril catalyzed secondary nucleation. **(B)** Radar plot illustrating the complementary effect of dimeric (red) and tetrameric (blue) S100B, under the conditions tested, on the diverse parameters illustrating Aβ42 aggregation and oligomer formation: secondary nucleation and fibril elongation rate constants (*k*_*2*_ and *k*_*+*_, respectively), maximum oligomer influx and total number of oligomers (measured, respectively, by peak height, PH, and area under the curve, AUC, of Aβ42 oligomerization distributions) and relative amounts of X-34 positive oligomers. To facilitate data comparison, each parameter was transformed by applying the operator *p* (–log_10_), such that a higher inhibition will result in a more eccentric point in the plot.

Our results revealed that dimeric and tetrameric S100B, the latter operating under sub-stoichiometric conditions and in the Ca^2+^ free state, are able to drastically decrease the reactive influx toward oligomers and AβO total amounts—as inferred by reductions in the peak height (PH) and area under curve (AUC) of oligomerization rate distributions, in addition to X-34 positive Aβ42 oligomers (Figure 5). We demonstrate that such inhibitory behavior is a consequence of a preferential suppression of fibril catalyzed nucleation (*k*_*2*_) of Aβ42 monomers into small aggregates (Figure 5A), in agreement with previous findings of an interaction between S100B and Aβ fibrils (Cristóvão et al., 2018; Figueira et al., 2022). With respect to fibril elongation, our results showed that although moderately, tetrameric apo-S100B is more competent than the dimer in suppressing the growth of aggregates (Figure 5B), a mechanism which constitute the main reactive sink for nucleated oligomers (Staats et al., 2020). We speculate that this might be relevant *in vivo*, where both S100B multimers co-exist (Ostendorp et al., 2007) and act concertedly to target multiple Aβ42 aggregation microscopic steps responsible for AβO formation and growth.

Altogether, our study sheds new insights on the catalytic landscape of the S100B chaperone, suggesting its critical role in the regulation of protein aggregation and neurotoxic oligomer formation in AD.

## Materials and methods

### Materials and proteins

All reagents were of the highest grade commercially available. A chelex resin (Bio-Rad, CA, USA) was used to remove contaminant trace metals from all buffers. Recombinant Aβ42 was expressed in *Escherichia coli* [BL21 (DE3) pLysS, Novagen] and purified as described (Walsh et al., 2009). To obtain the monomeric form, about 3 mg of Aβ42 was dissolved in 7 M guanidine hydrochloride (Sigma, MO, USA) and eluted in a Superdex S75 (GE Healthcare, IL, USA) with 50 mM HEPES [4-(2-hydroxyethyl)-1-piperazineethanesulfonic acid, NZYtech, Lisbon, Portugal] pH7.4. Monomeric Aβ42 concentration was estimated by UV spectroscopy (SPECTROstar Nano BMG LabTech, Ortenberg, Germany) at 280 nm using the theoretical extinction coefficient value of ε_280__nm_ = 1,424 M^–1^cm^–1^. Pre-formed fibrils (seeds) were prepared by incubating freshly isolated monomeric Aβ42 diluted in 50 mM HEPES pH7.4 for at least 24 h at 37°C. Fibril concentration was defined as monomer equivalents. Low-binding tubes (Axygen Scientific, Corning, NY, USA) were used in all manipulations of Aβ42. Human dimeric and tetrameric S100B were also expressed in *E. coli* [BL21 (DE3) E. Cline Express, Lucigen] and purified to homogeneity as described (Ostendorp et al., 2005; Botelho et al., 2012). S100B concentrations were estimated as homodimer equivalents using by UV spectroscopy at 280 nm using the theoretical extinction coefficient value of ε_280__nm_ = 2,980 M^–1^cm^–1^. Biochemical characterization and oligomeric state validation of S100B dimer and tetramer preparations was performed by size-exclusion chromatography and electrophoresis analysis as described (Supplementary Figure 5; Figueira et al., 2022).

### Aggregation kinetics

Aβ42 aggregation kinetics were performed by recording ThT (440 nm excitation filter/480 nm emission filter) or X-34 (370 nm excitation filter/480 nm emission filter) fluorescence intensity as a function of time in a plate reader (FLUOstar Optima, BMG Labtech, Ortenberg, Germany). The fluorescence was measured using bottom optics in half-area 96-well polyethylene glycol-coated black polystyrene plates with a clear bottom (Corning, 3881, NY, USA). The microplates were sealed with foil to avoid evaporation. Monomeric Aβ42 was diluted in 50 mM HEPES pH7.4 supplemented with 1.1 mM CaCl_2_ (Sigma) or 1.1 mM EDTA (ethylenedinitrilotetraacetic acid, Sigma, MO, USA) and the specified concentrations of dimeric/tetrameric S100B and pre-formed fibrils (seeds). ThT (*Twofold* Aβ42 monomer excess, Sigma, MO, USA) or X-34 (2 μM, Sigma, MO, USA in 100% dimethyl sulfoxide) was added to each condition. Appropriate controls in the absence of Aβ42 were performed to rule out the formation of ThT and X-34 positive species by S100B alone under identical experimental conditions (Supplementary Figure 6). All assays were performed in triplicates at 37°C, under quiescent conditions and fluorescence measurements taken every 400 s. Sample-size and descriptive statistical methods (mean ± standard deviation) were determined based on previous studies employing analogous *in vitro* Aβ42 aggregation assays (Cohen et al., 2013, 2015; Cristóvão et al., 2018).

### Transmission electron microscopy

For the analysis of structure and morphology of samples at different Aβ42 aggregation time-points, 5 μL aliquots directly removed from the aggregation plate were adsorbed into carbon-coated collodion film supported on 300-mesh copper grids (Electron Microscopy Sciences, PA, USA) and negatively stained twice with 1% (m/v) uranyl acetate (Electron Microscopy Sciences, PA, USA). Grids were visualized with a JEOL (Tokyo, Japan) JEM-1400 transmission electron microscope equipped with an Orious (CA, USA) Sc1000 digital camera, and exhaustively observed.

### Mechanistic analysis and simulations

Fitting of aggregation kinetics and rate constant estimation were performed on the AmyloFit (Meisl et al., 2016) online platform. ThT-monitored Aβ42 fibrillation kinetics in the absence or presence of S100B dimer/tetramer were globally fitted to the secondary nucleation dominated model which can by mathematically defined by the following set of differential equations (Eq. 1 and 2), expressing the time evolution of fibril number concentration [*P*(*t*)] and fibril mass concentration [*M*(*t*)] (Meisl et al., 2016).


(1) $$\frac{d\,P\,\left(t\right)}{d\,t}=k_{n}\,m\,{\left(t\right)}^{n_{c}}+k_{2}\,m\,{\left(t\right)}^{n_{2}}\,M\,\left(t\right)$$

(2) $$\frac{d\,M\,\left(t\right)}{d\,t}=2\,k_{+}\,m\,\left(t\right)\,P\,\left(t\right)$$

Where *k*_*n*_, *k*_*2*_ and *k*_*+*_ denote, respectively, the individual rate constants associated with primary nucleation, fibril catalyzed secondary nucleation and fibril elongation, *n*_*c*_ and *n*_*2*_ the reaction orders for primary and secondary nucleation (for Aβ42, *n*_*c*_ = *n*_2_ = 2) (Arosio et al., 2015) and *m*(*t*) the monomer concentration. Such equations can be solved in order to obtain the integrated rate law expressing the time progression of fibril mass [*M*(*t*)], as in Meisl et al. (2016).

The relative values of *k*_*+*_ in the absence and presence of S100B were determined from the linear fit of high-seeded (20%) kinetics at initial time-points. Under such conditions, Aβ42 aggregation is completely dominated by the elongation of pre-existing fibrils, exhibiting a pseudo-1st order hyperbolic kinetic whose initial slope is directly proportional to *k*_*+*_ (Meisl et al., 2014), according to the relation *v*_0_ = 2*k*_+_*m*(0)*P*(0). To constrain the number concentration of employed seeds [*P*(0)], the same fibril veil was used in all tested conditions. Aβ42 reactive flux toward oligomers [*r*(*t*)] were calculated in PLAS (Power Law Analysis and Simulation) (Voit, 2000) using the relative individual rate constants calculated in AmyloFit and taking in account the generation of new aggregates by primary and fibril-catalyzed secondary nucleation (Eq. 3). The total amounts of AβO/nucleation units were evaluated by integrating *r*(*t*) in respect to time.


(3) $$r\,\left(t\right)=k_{n}\,m\,{\left(t\right)}^{n_{c}}+k_{2}\,m\,{\left(t\right)}^{n_{2}}\,M\,\left(t\right)$$

Aβ42 oligomers normalized kinetic profile [*O*(*t*)] estimated from ThT-monitored aggregation rate constants was also computed in PLAS according to the reaction network and system of ODE (ordinary differential equations) depicted in Supplementary Figure 3.

## Data availability statement

The original contributions presented in this study are included in the article/Supplementary material, further inquiries can be directed to the corresponding author.

## Author contributions

CG conceived, designed, and supervised the study, analyzed the data, and wrote the manuscript with AF. AF, JS, and IC designed, conducted, and analyzed the experiments. All authors revised and approved the manuscript.
