# Supplementary material for: S100B chaperone multimers suppress the formation of oligomers during Aβ42 aggregation
Source: Front Neurosci. 2023 Mar 21;17:1162741. doi: 10.3389/fnins.2023.1162741 (PMC10070764; doi:10.3389/fnins.2023.1162741)
Supplement: Supplementary file 1 [file Data_Sheet_1.PDF]

Supplementary Materials for

S100B chaperone multimers suppress the formation of oligomers during Aβ42 aggregation

António J. Figueira[1,2], Joana Saavedra[3,4,5], Isabel Cardoso[3,4,5], Cláudio M. Gomes[1,2*]

[1] BioISI – Instituto de Biosistemas e Ciências Integrativas, Faculdade de Ciências, Universidade de Lisboa, 1749-016 Lisboa, Portugal
[2] Departamento de Química e Bioquímica, Faculdade de Ciências, Universidade de Lisboa, 1749-016 Lisboa, Portugal
[3] i3S – Instituto de Investigação e Inovação em Saúde, Universidade do Porto, Porto, Portugal
[4] IBMC - Instituto de Biologia Molecular e Celular, Universidade do Porto, Porto, Portugal
[5] ICBAS – Instituto de Ciências Biomédicas Abel Salazar, Universidade do Porto, Portugal

* Corresponding author: C.M. Gomes (cmgomes@fc.ul.pt), Telephone: (+351) 217 500 971

Supplementary Figure 1

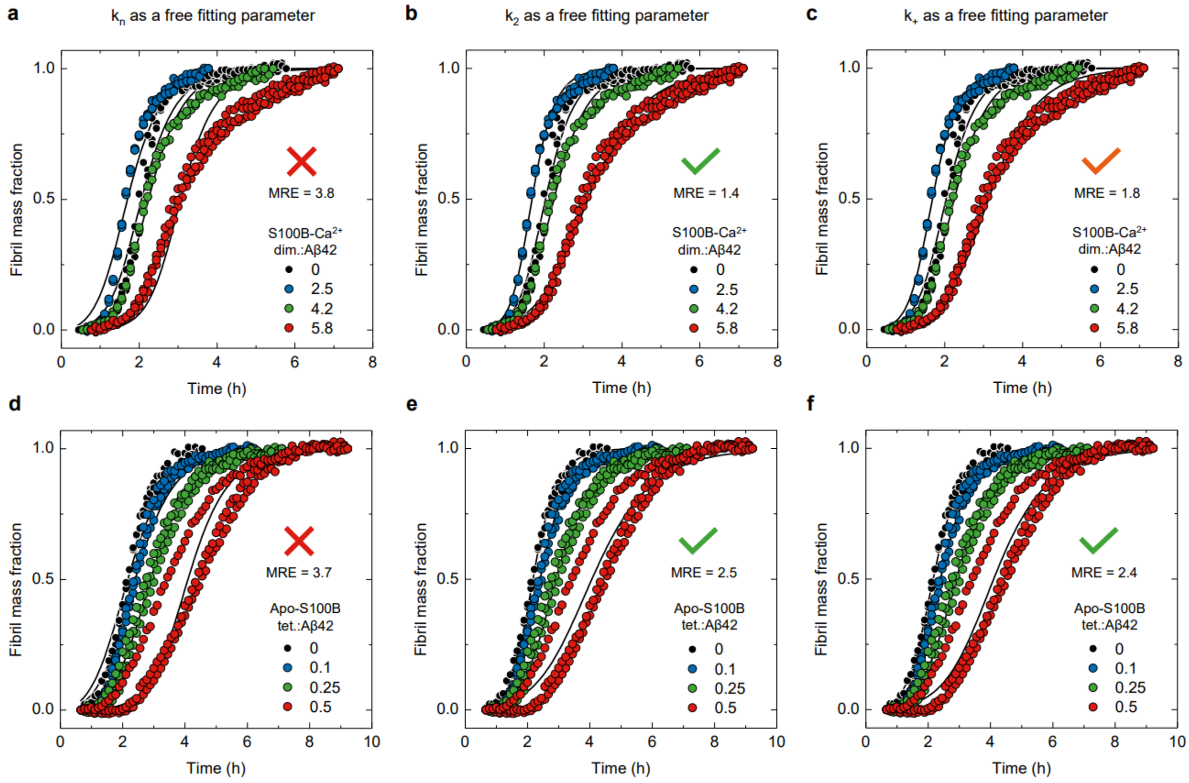

Supplementary Figure 1 – Global fitting of Aβ42 aggregation traces in the presence of different S100B dimer and tetramer molar-ratios allowing only a specific rate constant k_n, k_2 or k_+ to be a free-fitting parameter. Normalized kinetic traces of ThT-monitored aggregation of monomeric Aβ42 (6 µM in panels (a-c) or 2 µM in panels (d-f)) in the presence of increasing **(a, b, c)** S100B-Ca^{2+} dimer:Aβ42 (0 to 5.8) or **(d, e, f)** apo-S100B tetramer:Aβ42 (0 to 0.5) molar ratios. Solid lines depict sigmoidal fits of each curve by allowing selective variations of the individual rate constants associated with **(a, d)** primary nucleation k_n, **(b, e)** surface-catalysed secondary nucleation k_2 and **(c, f)** fibril elongation k_+. In all cases, the Mean Residual Error (MRE) describing the fitting quality of three experiments per condition is presented.

Supplementary Figure 2

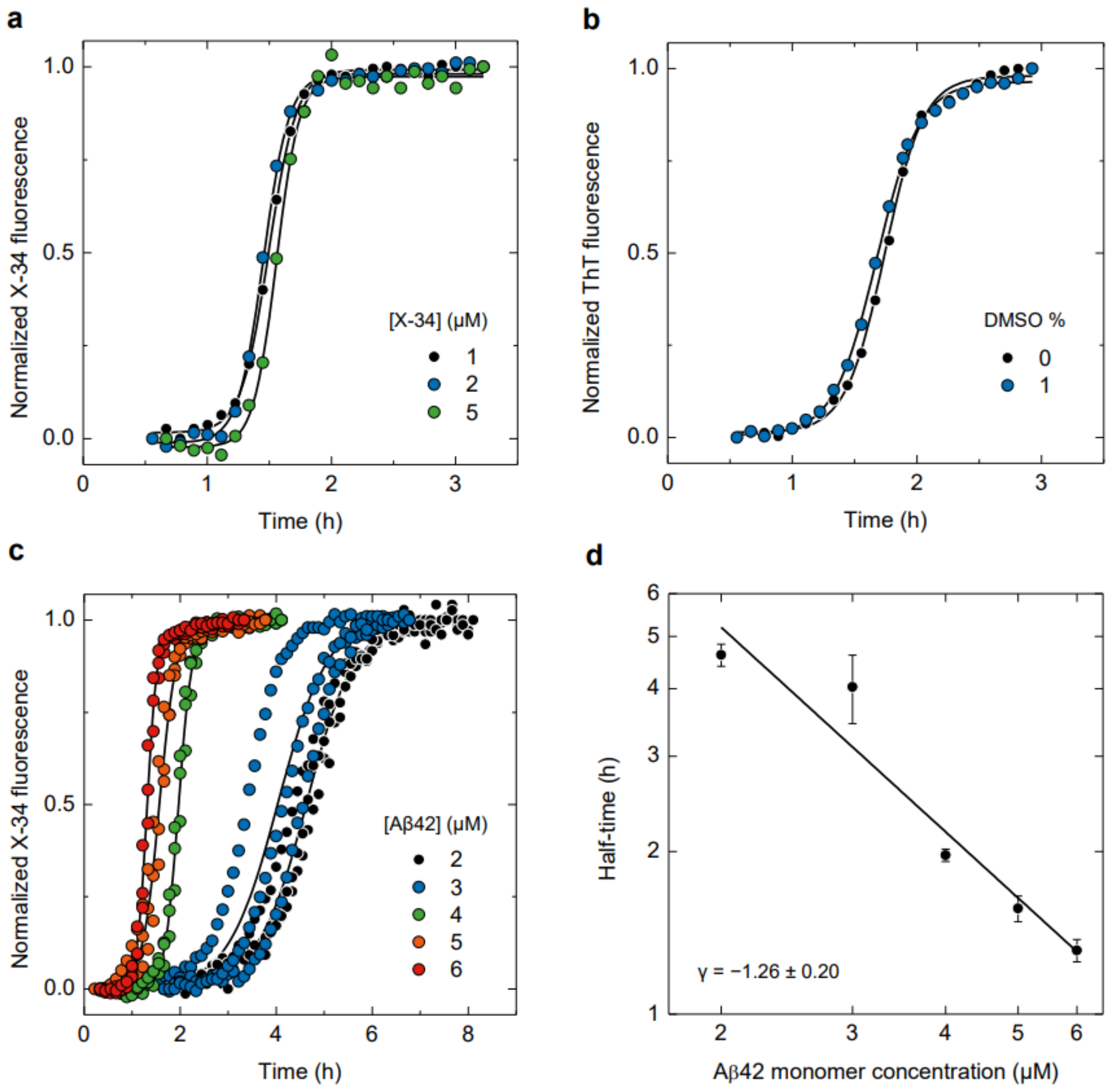

Supplementary Figure 2 – X-34 monitored Aβ42 aggregation controls and scaling exponent analysis. (a) Normalized kinetic traces Aβ42 (6 µM) aggregation monitored at different concentration of X-34 (1, 2 and 5 µM). **(b)** Normalized kinetic traces Aβ42 (6 µM) aggregation monitored by ThT in the absence and presence of 1% (v/v) dimethyl sulfoxide (DMSO). **(c)** X-34 monitored Aβ42 aggregation at increasing monomer concentrations (2 to 6 µM) and **(d)** determination of Aβ42 dominant nucleation mechanism by analysis of scaling exponent value (γ) calculated by the slope of the double-log plot relating monomer concentrations and aggregation half times. Error bars represent standard deviation of three experiments.

Supplementary Figure 3

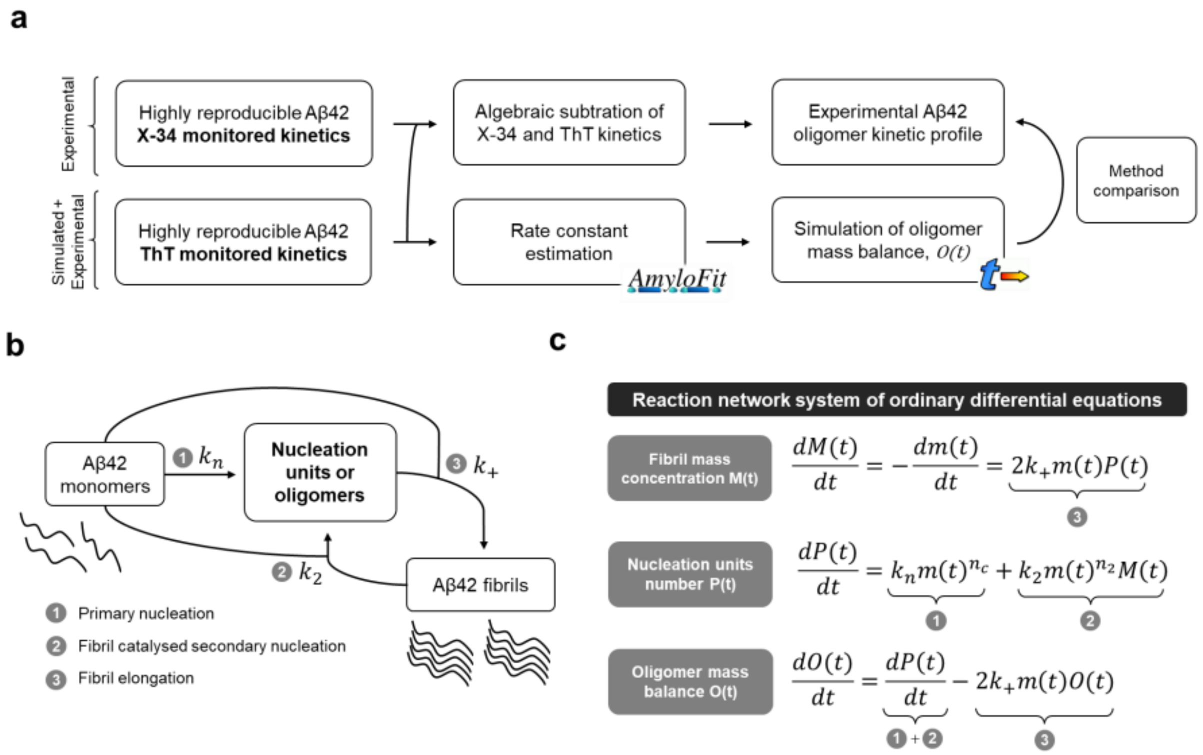

Supplementary Figure 3 – Aβ42 oligomer mass ($\boldsymbol{O(t)}$) simulation from ThT-monitored aggregation assays. (a) Simplified workflow employed to access X-34 selectivity for Aβ42 oligomer detection. Briefly, we compared ThT-subtracted X-34 kinetic profiles with the temporal evolution of nucleation units derived from ThT-monitored aggregation rate constants. We modelled Aβ42 aggregation by **(b)** a simple reaction network which recapitulates the main mechanisms described to govern Aβ42 *in vitro* fibrillation, namely primary/fibril catalysed nucleation of monomers into small aggregates and fibril elongation. **(c)** The corresponding system of ordinary differential equations was then generated and numerically solved in PLAS using AmyloFit calculated rate constants (k_n, k_2 and k_+), in order to access the temporal variation of Aβ42 on-pathway oligomer mass fraction ($O(t)$).

Supplementary Figure 4

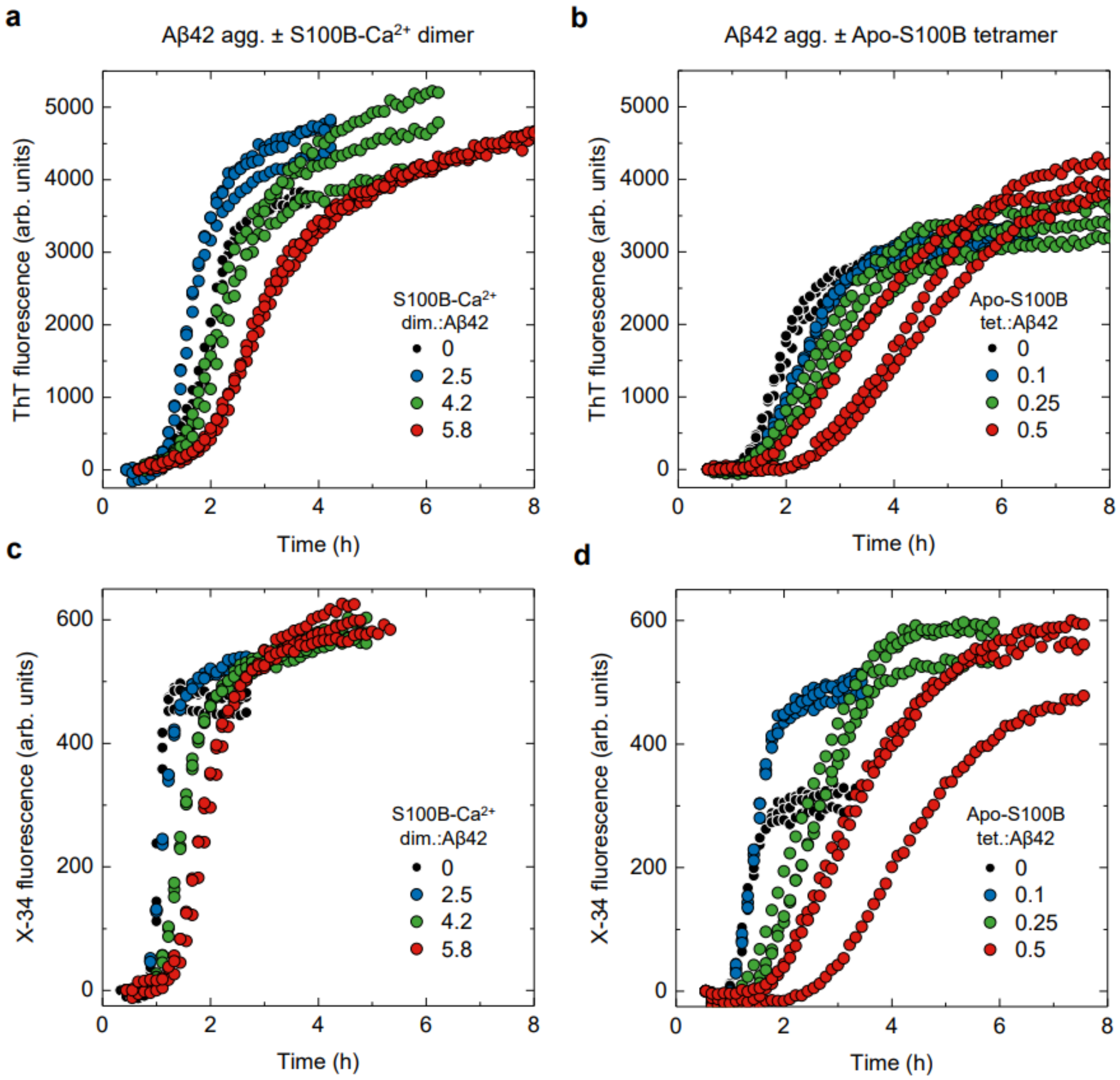

Supplementary Figure 4 – Non-normalized traces of ThT and X-34 monitored Aβ42 aggregation under the conditions depicted in Figure 4. Non-normalized kinetic traces of **(a, b)** ThT-monitored and **(c, d)** X-34-monitored aggregation of monomeric Aβ42 (6 µM in panels (a, c) and 2 µM in panels (b, d) in the presence of increasing **(a, c)** S100B-Ca^{2+} dimer:Aβ42 (0 to 5.8) or **(b, d)** apo-S100B tetramer:Aβ42 (0 to 0.5) molar ratios.

Supplementary Figure 5

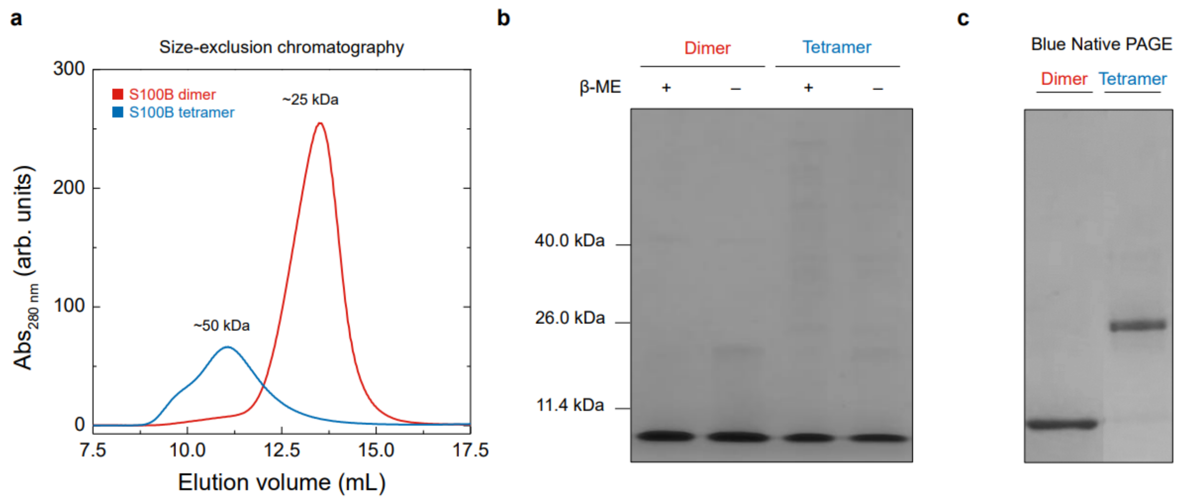

Supplementary Figure 5 – Characterization of the isolated recombinant S100B multimers. (a) Confirmation of S100B oligomeric state by size-exclusion chromatography: dimer (red, ~25 kDa) and tetramer (blue, ~50 kDa). **(b)** SDS-PAGE analysis of S100B oligomers under reducing (+ β-ME) and non-reducing (– β-ME) conditions to demonstrate that the obtained S100B dimer and S100B tetramers do not involve S-S crosslinks. Both dimeric and tetrameric S100B migrate as a single band near the gel front, corresponding to the denatured monomer (10.7 kDa); β-ME, β-mercaptoethanol. **(c)** Blue Native PAGE assessment of S100B oligomeric state of dimeric and tetrameric fractions to confirm sample homogeneity; the S100B tetramer band displays lower electrophoretic mobility, in agreement with a higher molecular mass.

Supplementary Figure 6

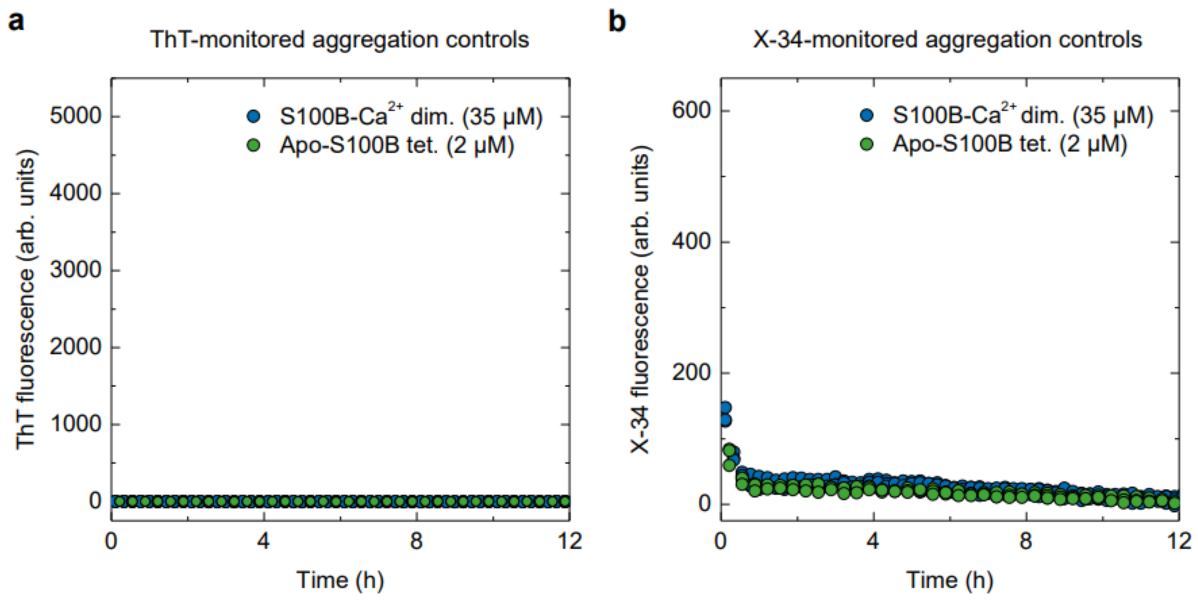

Supplementary Figure 6 – Control experiments showing that no (a) ThT or (b) X-34 positive species are formed by either S100B-Ca^{2+} dimer (35 μM) or apo-S100B (2 μM) tested at the highest S100B concentrations used in the study.
